# Comparison of esmolol and labetalol, in low doses, for attenuation of sympathomimetic response to laryngoscopy and intubation

**DOI:** 10.4103/1658-354X.71573

**Published:** 2010

**Authors:** Sarvesh P. Singh, Abdul Quadir, Poonam Malhotra

**Affiliations:** *Department of Anaesthesia, J N Medical College, Aligarh, India*

**Keywords:** *Labetalol*, *esmolol*, *sympathomimetic*, *response*, *intubation*

## Abstract

**Objective:**

The present study compared the efficacy of esmolol and labetalol, in low doses, for attenuation of sympathomimetic response to laryngoscopy and intubation.

**Design:**

Prospective, randomized, placebo controlled, double-blinded study.

**Setting:**

Operation room.

**Patients and Methods:**

75 ASA physical status I and II adult patients, aged 18-45 years undergoing elective surgical procedures, requiring general anesthesia and orotracheal intubation.

**Interventions:**

Patients were allocated to any of the three groups (25 each)-Group C (control)10 ml 0.9% saline i.v. Group E (esmolol) 0.5 mg/kg diluted with 0.9% saline to 10 ml i.v. Group L (labetalol) 0.25 mg/kg diluted with 0.9% saline to 10 ml i.v. In the control group 10 ml of 0.9% saline was given both at 2 and 5 min prior to intubation. In the esmolol group 0.5 mg/kg of esmolol (diluted with 0.9% saline to 10 ml) was given 2 min prior and 10 ml of 0.9% saline 5 min prior to intubation. In the labetalol group 10 ml of 0.9% saline was administered 2 min prior and 0.25 mg/kg of labetalol (diluted with 0.9% saline to 10 ml) 5 min prior to intubation. All the patients were subjected to the same standard anesthetic technique.

**Measurements:**

Heart rate (HR), systolic blood pressure (SBP) and diastolic blood pressure (DBP) were recorded prior to induction, at time of intubation and 1, 3, 5, and 10 min after intubation. Mean arterial pressure (MAP) and rate pressure product (RPP) were calculated. Abnormal ECG changes were also recorded.

**Results:**

Compared to placebo and esmolol (0.5 mg/kg), labetalol (0.25 mg/kg) significantly attenuated the rise in heart rate, systolic blood pressure, and RPP during laryngoscopy and intubation. However, the difference was not statistically significant among the values for DBP and MAP.

**Conclusion:**

In lower doses, labetalol (0.25 mg/kg) is a better agent than esmolol (0.5 mg/kg) in attenuating the sympathomimetic response to laryngoscopy and intubation.

## INTRODUCTION

Despite the emergence of new airway devices in the recent years, rigid laryngoscopy and tracheal intubation still remain the gold standard in airway management. The hemodynamic changes stemming from airway instrumentation are due to sympathoadrenal discharge caused by epipharyngeal and parapharyngeal stimulations. There is increase in heart rate (HR), blood pressure, intraocular, and intracranial pressure. The arterial hypertension is due to increase in cardiac output rather than an increase in systemic vascular resistance, and is associated with the transient rise in central venous pressure. Arrhythmias also tend to occur.

The major conditions in which these responses may pose serious challenges are cardiovascular diseases like hypertension, coronary artery disease, aneurysmal vascular disease or those with decreased intracranial compliance like head injury with extra or intradural hematoma formation, intracranial tumors, etc. A sudden rise in blood pressure may cause left ventricular failure, myocardial ischemia, and cerebral hemorrhage. These complications are more likely in the presence of coronary or cerebral atheroma or hypertension. Also convulsions may be precipitated in pre-eclamptic patients.

Various attempts have been made to suppress this pressor response. The pharmacological methods are aimed at efferent, afferent, or both limbs of response e.g. volatile inhalational agents,[1] lignocaine,[2] opioids,[3] sodium nitroprusside,[4] nitroglycerine[5] calcium channel blockers,[6] and adrenergic blockers.[7]

Most workers have used esmolol[8–12] (cardioselective beta blocker) as a bolus and in infusion and found it to be effective. Other beta blockers like metoprolol[13,14] and labetalol[15–18] have been useful in not only attenuating the response of laryngoscopy and intubation but also in preventing perioperative cardiovascular events. However studies comparing esmolol with labetalol (non selective adrenergic blocker) as an attenuating agent for pressor response are lacking.

## PATIENTS AND METHODS

### Study design

This study was a prospective, randomized, placebo controlled, double-blinded trial comparing two adrenergic antagonists labetalol (nonselective) and esmolol (B1 selective) in decreasing the pressor response during rigid laryngoscopy and intubation. The protocol was approved by the Institutional Review Board and was in accordance with International Conference on Harmonisation; Good Clinical Practice (ICH-GCP) standards.

### Duration of study

March 2006 - August 2007

### Sample

Sample size was calculated by power analysis, using a two-sample *t* test, with a two-sided type I error of 5% (α=0.05) and power at 80.37 (α=0.19). Therefore, 75 patients, ASA physical status I and II, aged 18-45 years, undergoing elective surgical procedures, requiring general anesthesia and orotracheal intubation were included in the study. Informed consent was obtained from all the patients. Patients with cardiovascular, pulmonary, hepatic, and renal disease; those on B blockers; patients with difficult airway; laryngoscopy and intubation time more than 30 s, or requiring more than two attempts were excluded from the study.

### Procedure

The patients were randomly (computer generated randomization schedule) allocated into one of the three groups, of 25 each. Blinding was done using the SNOSE (sequentially numbered opaque sealed envelope) technique. Patients were allocated to any of the three groups-

**Table:** 

Group C (control)	10 ml 0.9% saline i.v.
Group E (esmolol)	0.5 mg/kg diluted with 0.9% saline to 10ml i.v.
Group L (labetalol)	0.25 mg/kg diluted with 0.9% saline to 10ml i.v.

In the control group 10 ml of 0.9% saline was given both at 2 and 5 min prior to intubation. In the esmolol group 0.5 mg/kg of esmolol (diluted with 0.9% saline to 10 ml) was given 2 min prior and 10 ml of 0.9% saline 5 min prior to intubation. In the labetalol group 10 ml of 0.9% saline was administered 2 min prior and 0.25 mg/kg of labetalol (diluted with 0.9% saline to 10 ml) 5 min prior to intubation.

Patients were kept nil orally for 8 h prior to surgery.

All patients were premedicated intravenously 10 min prior to induction with inj. ondansetron 0.1 mg/kg, inj. tramadol 2 mg/kg, and inj. midazolam 0.05 mg/kg.

The patients were pre-oxygenated with 100% 02 by a face mask for 3 min. Induction was done with inj. thiopentone 5 mg/kg and after 30 s relaxation achieved with inj rocuronium bromide 1 mg/kg. 90s later the patient was intubated using a Macintosh laryngoscope. All intubations were done by the same experienced laryngoscopist. Tracheal tubes of ID 7.0 mm and 8.0 mm were used for female and male patients, respectively. Anesthesia was maintained by N2O (60%) and O_2_ (40%), intermittent boluses of vecuronium bromide intravenously and propofol infusion (5 mg/kg/hr). At the end of surgery, neuromuscular blockade was reversed with inj. neostigmine (40 μg/kg) and inj. atropine (20 μg/kg).

### Measures

HR, systolic blood pressure (SBP), and diastolic blood pressure (DBP) were recorded prior to induction, at time of intubation and 1, 3, 5, and 10 min after intubation. Mean arterial pressure (MAP) and rate pressure product (RPP) were calculated for the same time stations. Abnormal ECG changes were also recorded.

### Statistics analysis

Statistical analysis was performed using the SPSS software version 13 (Chicago, IL, USA). Patient demographics were compared with analysis of variance (ANOVA). The study data were analyzed using statistical methods of mean, standard deviation, paired students “*t*” test (for values within the group at different time stations) and independent samples “*t*” test (for comparison of intergroup values).

## RESULTS

The patients in the three groups were comparable with respect to age, weight, sex, and duration of surgery or anesthesia [Table 1].

**Table 1:** Demographic data

	Group C	Group E	Group L
Mean age (Yrs)	30.28	31.08	30.56
Weight (Kg)	58.1	56	57.8
Male/Female	17/8	15/10	15/10

The preinduction values of pulse rate (PR) were comparable between groups with no significant difference [Table 2].There was no statistically significant difference in PR throughout study time between the esmolol and control groups (*P*>0.05). At intubation and 1 min postintubation PR was significantly lower in the labetalol group compared to the control group (*P*<0.001and *P*=0.012 respectively). At 3 and 5 min postintubation, there was no significant difference in PR (*P*=0.17 and *P*=0.37 respectively) between labetalol and control groups. At 10^th^ minute PR was significantly lower in the labetalol group than the control group (*P*<0.001). The PR were significantly less in the labetalol group throughout the study time compared with the esmolol group (<0.001 at intubation and 1^st^ minute postintubation, *P*=0.02 at 3^rd^ minute, *P*=0.01 at 5^th^ and *P*<0.001 at 10^th^ minute postintubation).

**Table 2:** Pulse rates

	Group C (/min)	Group E (/min)	Group L (/min)	*P* value C and E	*P* value C and L	*P* value E and L
Preinduction	81.16± 9.55	85.76±8.33	85.24±14.26	0.076	0.241	0.876
At intubation	114.76±15.19*	108.64±11.41*	97.4±9.01*	0.114	<0.001	<0.001
Postintubation						
1 min	107.44±14.15*	109.64±8.57*	98.16±10.6*	0.509	0.012	<0.001
3 min	94.68±9.92*	97.44±9.17*	90.56±11.03	0.312	0.172	0.020
5 min	87.88±9.03*	93.68±12.25*	85.28±11.35	0.063	0.375	0.015
10 min	85.32±6.94*	90.16±11.23	76.16±7.76*	0.073	<0.001	<0.001

Mean values±SD.,

* *P*<0.05 within group (vs preinduction value)

The preinduction values of SBP were comparable between groups with no significant difference [Table 3]. SBP increased in both esmolol and control groups at all times. However, no significant difference was present between the groups (*P*>0.05). Compared with the control group values [Table 3] SBP was significantly lower at all time stations in the labetalol group (*P*<0.001 at intubation, 1^st^ and 3^rd^ minute postintubation; *P*=0.004 at 5^th^ minute and *P*=0.02 at 10^th^ minute postintubation). SBPs were significantly less in patients receiving labetalol compared to those who received esmolol (*P*<0.01 at intubation and 1, 3 and 10 min postintubation and *P*=0.014 at 5 min postintubation).

**Table 3:** Systolic blood pressures

	Group C (mm Hg)	Group E (mm Hg)	Group L (mm Hg)	*P* value C and E	*P* value C and L	*P* value E and L
Preinduction	122.8±9.88	121.04±9.14	126.08±10.57	0.517	0.263	0.078
At intubation	162.4±14.3*	154.24±17.35*	140.72±16.99*	0.076	<0.001	0.008
Postintubation						
1 min	156.08±14.3*	158.72±16.77*	139.36±12.56*	0.552	<0.001	<0.001
3 min	140.48±12.37*	137.44±16.44*	125.04±11.33	0.464	<0.001	0.003
5 min	127.36±10.85*	127.36±14.62*	118.32±10.12*	1.000	0.004	0.014
10 min	120.96±10.49	121.68±9.12	113.68±11.3*	0.797	0.022	0.008

Mean values±SD

* *P*<0.05 within group (vs preinduction value)

The preinduction values of DBP were comparable between groups with no significant difference [Table 4]. Table 4 shows that DBP at 1 minute postintubation in the esmolol group was significantly less than that in the control group (*P*=0.028). At all other times it was comparable between the groups (*P*>0.05). DBP in the labetalol group was comparable with the control group with no significant difference. Diastolic pressures were not significantly different between labetalol and esmolol groups (*P*>0.05).

**Table 4:** Diastolic blood pressures

	Group C (mm Hg)	Group E (mm Hg)	Group L (mm Hg)	*P* value C and E	*P* value C and L	*P* value E and L
Preinduction	78.64±5.34	79.12±6.9	81.28±5.82	0.770	0.076	0.238
At intubation	102.08±6.86*	98.8±6.95*	100.64±13.67*	0.100	0.640	0.551
Postintubation						
1 min	99.12±6.53*	94.96±6.48*	97.52±9.92*	0.028	0.604	0.286
3 min	90.96±7.00*	87.36±6.23*	89.76±6.61*	0.061	0.536	0.193
5 min	85.20±7.09*	82.40±6.29*	82.80±9.11	0.147	0.304	0.857
10 min	79.12±4.83	78.96±6.80	79.04±7.53	0.924	0.965	0.969

Mean values±SD

* *P*<0.05 within group (vs preinduction value)

The preinduction values of MAP were comparable between groups with no significant difference [Table 5]. MAP was significantly less at the time of intubation in the esmolol group (*P*<0.05) compared with the control group. All other postintubation values were comparable between the two groups and not statistically significant (*P*>0.1). Compared with controls [Table 5], it was significantly less in the labetalol group at all times except at 10^th^ minute postintubation (*P*=0.012 at intubation, *P*<0.01 at 1^st^ and 3^rd^ minute postintubation, *P*=0.04 at 5^th^ minute and *P*=0.22 at 10^th^ minute postintubation). There was no statistically significant difference between values of labetalol and esmolol groups (*P*>0.15), except at 1 min postintubation when it was significantly less in the labetalol group (*P*=0.042).

**Table 5:** Mean arterial pressures

	Group C (mm Hg)	Group E (mm Hg)	Group L (mm Hg)	*P* value C and E	*P* value C and L	*P* value E and L
Preinduction	93.36±5.50	93.09±7.03	96.21±5.80	0.882	0.081	0.094
At intubation	122.18±8.24*	117.28±8.53*	114.00±13.33*	0.044	0.012	0.305
Postintubation						
1 min	118.10±7.74*	116.21±6.90*	111.46±9.01*	0.363	0.007	0.042
3 min	107.46±7.66*	104.05±8.09*	101.52±6.71*	0.132	0.005	0.234
5 min	99.25±7.49*	97.38±8.08*	94.64±8.36	0.402	0.046	0.244
10 min	93.06±5.89	93.2±6.74	90.58±8.12*	0.940	0.223	0.222

Mean values±SD

* *P*<0.05 within group (vs preinduction value)

The preinduction values of RPP were comparable between groups with no significant difference [Table 6]. RPP was significantly less at the time of intubation in the esmolol group (*P*<0.05) as compared to the control group. All other postintubation values were comparable between the groups and not statistically significant (*P*>0.1). RPP values were lower in the labetalol group compared to the control group [Table 6]. The difference was statistically significant at all times (*P*<0.001 at intubation, 1^st^, 3^rd^ and 10^th^ minute postintubation and *P*=0.03 at 5^th^ minute postintubation). RPP was significantly lower in the labetalol group at all times (*P*<0.001 at intubation and 1 and 10 minutes postintubation and *P*<0.01 at 3^rd^ and 5^th^ minute postintubation). The RPP in the labetalol group never crossed the critical 15000 mark.

**Table 6:** Rate pressure products

	Group C (mm Hg /min)	Group E (mm Hg /min)	Group L (mm Hg /min)	*P* value C and E	*P* value C and L	*P* value E and L
Preinduction	9936±1159	10420±1562	10726±1889	0.220	0.081	0.535
At intubation	18689±3059*	16894±3203*	13719±2136*	0.048	<0.001	<0.001
Postintubation						
1 min	16676±2315*	17471±2746*	13647±1769*	0.275	<0.001	<0.001
3 min	13241±1295*	13438±2325*	11336±1846	0.714	<0.001	0.001
5 min	11154±1340*	11995±2468*	10143±1938	0.141	0.037	0.005
10 min	10289±893*	11017±1957*	8680±1401*	0.097	<0.001	<0.001

Mean values±SD

* *P*<0.05 within group (vs preinduction value)

Three episodes of atrial ectopics were recorded just after intubation in control (two episodes: at time of intubation and 3 min postintubation) and esmolol groups (1 min postintubation). Twenty eight (7 of 25) percent patients in the labetalol group developed bradycardia (pulse rate<50 beats per minute) after study period of 10 min.

## DISCUSSION

Most of the clinicians use adjuncts to attenuate the sympathetic response associated with laryngoscopy and intubation in high risk patients. Beta blockers have been compared with fentanyl,[3] nitroprusside,[4] nitroglycerine,[5] calcium channel blockers,[6] etc; however, studies comparing esmolol[8–12] (cardioselective beta blocker) and labetalol[15,18] (nonselective adrenergic blocker) are lacking.

Esmolol hydrochloride is an ultra-short acting, beta-one selective adrenergic receptor blocker with a distribution half-life of 2 min and an elimination half-life of 9 min. Esmolol appears quite suitable for use during a short-lived stress such as tracheal intubation or ECT. Labetalol is an adrenergic receptor blocking agent with mild alpha1- and predominant beta-adrenergic receptor blocking actions (alpha:beta blockade ratio of 1:7 for iv and 1:3 for PO administration). The onset of action of i.v. labetalol is 5 min.

We studied the hemodynamic response to laryngoscopy and intubation for a period of 10 min as this is the average period for which hemodynamic changes are believed to last.[19]

There was no significant effect of esmolol on PR when compared to the control group. Labetalol had a significantly (*P*<0.05) better effect than esmolol in controlling PR at all points during the study. It seems that when instrumentation stimulus is present labetalol maintains the PRs within normal ranges. When the effect of stimulus weans off, as occurs at 10 min postintubation, the drug’s effect takes over and pulse rates go below baseline values.

In preventing the increases in SBP esmolol was completely ineffective as there was no significant difference between values of esmolol and control groups during the study period (*P*>0.05). Labetalol prevented the increase in SBP significantly throughout the study period as compared to control and esmolol groups (*P*<0.05). Ramanathan *et al*.[18] used 20 mg labetalol to prevent rise in SBP successfully. Inada *et al*.[17] found 10 mg (0.14 mg/kg) labetalol ineffective in attenuating the rise in systolic pressure. This difference might be because of the lower dose they used and the timing of giving of labetalol (2 min prior to intubation) because of which the peak effect of drug was lost at intubation. Maharaj *et al*.[20] failed to blunt the blood pressure response with 0.25 and 0.5 mg/kg labetalol. However, they did not mention the timing of giving the drug. Esmolol even in doses exceeding >1mg/kg have been found to be ineffective in controlling systolic pressure rise.

When compared to controls the rise in DBP was not attenuated (*P*>0.05) in any of the study groups [Table 4]. There was a significant difference between esmolol and control values at 1 minute postintubation (*P*<0.05). This was an isolated finding because no significant difference was observed at subsequent points of study. In intergroup comparison of esmolol and labetalol, none of them was found to be better (*P*>0.05).

Comparing the esmolol group with controls [Table 5] revealed that the esmolol group had a significantly less MAP at intubation (*P*=0.044). This observation is the same as made by Sharma *et al*.[8] and Bakiye *et al*.[21] in their studies, although esmolol was not at all effective in controlling MAP rise after laryngoscopy and intubation (*P*>0.05). When the labetalol group was compared with the control group the MAP was significantly less at all points (*P*<0.05) except at 10 min postintubation when the values were comparable. Between esmolol and labetalol there was no significant difference in values except at 1 min postintubation (labetalol having lower MAPs). This observation was again an isolated finding and no significant difference (*P*>0.05) was found at any other point during the study period. MAP increase was attenuated by labetalol but not esmolol.

In esmolol-treated patients RPP was not significantly different from the control group. Compared to control and esmolol groups, the labetalol group had significantly lower values of RPP [Table 6]. Labetalol could not prevent the increase in RPP completely (significantly elevated at intubation and at 1 min postintubation). However, the magnitude of increase was less and never crossed the critical limit of 15000 mmHg/min. The values returned to baseline at 3 min postintubation as compared to other groups where they achieved baseline values after 10 min. Therefore, labetalol (0.25 mg/kg) decreases the magnitude and duration of hemodynamic response to laryngoscopy as evident from changes of RPP. Leslie *et al*.[22] used labetalol in doses of 0.25, 0.5, 0.75 and 1.0 mg/kg and found all doses effective in controlling the rise in RPP at laryngoscopy.

The only side effect observed was that of labetalol in form of bradycardia, intraoperatively. Seven patients (28 %) developed bradycardia (pulse rate <50 beats per minute) after the study period of 10 min and had to be given atropine in 0.2 mg increments (max. 0.01 mg/kg). All the patients responded to atropine treatment. There were no recurrent episodes of bradycardia. No other side effects were observed. We recorded three episodes of atrial ectopics just after intubation. The atrial ectopics recorded in our study were attributed to tracheal intubation and not thiopentone induction.[23,24] All three ectopics occurred at the time of intubation or just after the intubation and there were no abnormal ECG changes between the duration of induction and intubation.

## CONCLUSION

In lower doses, labetalol (0.25 mg/kg) is a better agent than esmolol (0.5 mg/kg) in attenuating the sympathomimetic response to laryngoscopy and intubation whereas low dose esmolol (0.5 mg/kg) is ineffective for the same purpose. Bradycardia is a potential side effect of labetalol.
